# Are nestin-positive mesenchymal stromal cells a better source of cells for CNS repair?

**DOI:** 10.1016/j.neuint.2016.08.001

**Published:** 2017-06

**Authors:** Susan L. Lindsay, Susan C. Barnett

**Affiliations:** Institute of Infection, Inflammation and Immunity, Glial Cell Biology Group, Sir Graeme Davies Building, Room B329, 120 University Place, University of Glasgow, Glasgow, G12 8TA, United Kingdom

**Keywords:** Human mesenchymal stromal cells, Bone marrow, Olfactory mucosa, Myelination, Nestin

## Abstract

In recent years there has been a great deal of research within the stem cell field which has led to the definition and classification of a range of stem cells from a plethora of tissues and organs. Stem cells, by classification, are considered to be pluri- or multipotent and have both self-renewal and multi-differentiation capabilities. Presently there is a great deal of interest in stem cells isolated from both embryonic and adult tissues in the hope they hold the therapeutic key to restoring or treating damaged cells in a number of central nervous system (CNS) disorders. In this review we will discuss the role of mesenchymal stromal cells (MSCs) isolated from human olfactory mucosa, with particular emphasis on their potential role as a candidate for transplant mediated repair in the CNS. Since nestin expression defines the entire population of olfactory mucosal derived MSCs, we will compare these cells to a population of neural crest derived nestin positive population of bone marrow-MSCs.

## Highlights

• Human olfactory mucosa is a new source of mesenchymal stromal cells (MSCs).
• Some bone marrow MSCs are nestin-positive, neural crest derived and regulate hematopoietic stem cell activation.
• Human olfactory mucosa contains a population of nestin-positive MSCs that secrete CXCL12 and may have promote CNS repair.

## 1 Introduction

Friedenstein was the first to identify that single cell suspensions of bone marrow (BM) stroma could generate colonies of adherent fibroblast-like cells *in vitro* (Friedenstein et al., 1968). These colony-forming unit fibroblasts (CFU-Fs) were found to be capable of osteogenic differentiation and provided the first evidence that clonogenic stem cell precursors existed of the bone lineage (Friedenstein et al., 1968, Friedenstein et al., 1970). Later these stromal cells were classified as stem cells, since single cells could transdifferentiate into multi-lineage cells of bone and osteogenic tissue (Friedenstein, 1980). Their eventual capability of generating the osteogenic, chondrogenic and adipogenic mesenchymal lineages meant they were then given the title of mesenchymal stem cells (Caplan, 1991, Fig. 1). It was also shown that whilst they cannot make hematopoietic stem cells (HSCs), they do physically support them and promote their differentiation (Dexter, 1982, Owen, 1988). Interestingly, Caplan discussed the concept of cell transplantation therapy using MSCs therapeutically, but as a source of bone and connective tissue (Caplan, 1991). This became more pertinent when it was shown that MSCs only express the class I major histocompatibility complex (MHC-1) but not class II or co-stimulatory molecules such as CD40, CD80 and CD86 making them less likely to raise an immune response (Le Blanc, 2003). It has also been suggested that due to their limited pluripotent potential, MSCs should be re-named and termed “mesenchymal stromal cells” to avoid the excessive promotion of their stem cell potential (Horwitz et al., 2005, Pacini and Petrini, 2014). Therefore, in this review the abbreviation MSC is referred to as mesenchymal stromal cells (MSCs).

**Fig. 1:**
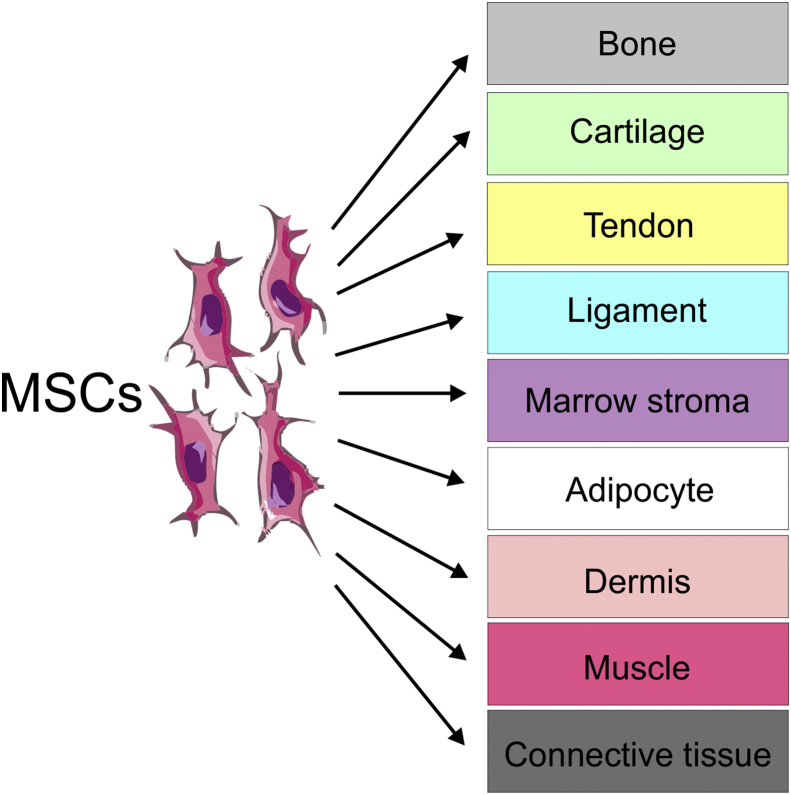
Differentiation of MSCs based on Caplan, 1991. MSCs have the capacity to differentiate into osteogenic, chondrogenic and adipogenic mesenchymal lineages.

### 1.1 MSCs and their origins

MSCs are known to be present in virtually all postnatal organs and tissues including heart, lung umbilical cord, peripheral blood, adipose tissue, muscle, cartilage, synovium, dental pulp, BM, tonsil, placenta, thymus and olfactory mucosa (OM) (da Silva Meirelles et al., 2006, Kuhn and Tuan, 2010; Lindsay et al., 2013, Xie et al., 2015, Lindsay et al., 2016). However, whether they permanently reside in such tissues, or can circulate in the blood or even exist in perivascular spaces to reach different tissues is still not known (Pacini and Petrini, 2014). By definition MSCs must i) adhere to plastic, ii) express specific cell surface markers and iii) differentiate in a multipotential manner along the osteogenic, chondrogenic, and adipogenic lineages (Dominici et al., 2006). A panel of markers are used to define MSCs including CD73 (ecto-5′nucleotidase) CD90 (Thy-1), CD105 (endoglin), CD166 (ALCAM), CD271 (p75NFGR/NTR), CD44 and STRO-1. However, none of these are specific and will also label a range of other cell types including endothelial cells, epithelial cells, fibroblasts, T cells and certain neural cell types (Kuhn and Tuan, 2010, Xie et al., 2015). MSCs also lack expression of CD34 (hematopoietic progenitor and endothelial cell marker), CD45 (pan-leukocyte marker), CD11b or CD14 (monocyte and macrophage markers), CD19 or CD79a (B cell markers), and HLA-DR (marker of stimulated MSCs) (Mo et al., 2016). Initially their purification from BM was carried out by differential adherence to plastic since only the MSCs from stroma will adhere. However, there are now specific isolation kits available based on cell surface antibodies and magnetic selection which can be used to highly enrich for MSCs from a variety of different tissue sources, including BM. To add to the complexity, MSCs share cell-surface markers and localisation with pericytes, making their true classification and distinction even more complex (Crisan et al., 2008). Importantly, in the context of their therapeutic potential, these cells are widely available, have a high capacity to self-renew and are easily propagated in culture in substantial enough numbers. However the lack of standardised protocols for their expansion and isolation makes results difficult to interpret (Pacini and Petrini, 2014).

### 1.2 MSCs from the human olfactory mucosa

The uniquely regenerative properties of the olfactory system (Graziadei and Monti Graziadei, 1983) has meant that this tissue has gained much interest for the transplant mediated repair of the CNS (Barnett and Riddell, 2007, Lindsay et al., 2010, Roet and Verhaagen, 2014, Tabakow et al., 2013). Some of the transplantation studies have incorporated the use of the entire OM, while others have used the purified glial cell population, known as olfactory ensheathing cells (Li et al., 1997, Ramón-Cueto et al., 2000). We undertook a study to identify the stem cell population(s) from this tissue, since many researchers were already transplanting cells from OM into patients (Lima et al., 2006, Mackay-Sim et al., 2008, Geraghty, 2008). We identified MSC-like cells from the lamina propria of the human OM using CD271 purification and selection, which we termed OM-MSCs (Lindsay et al., 2013, Johnstone et al., 2015). Detailed comparison was made with classical BM-derived MSCs which were isolated and maintained using identical methods and culture conditions (Lindsay et al., 2013, Johnstone et al., 2015). We demonstrated that the OM-MSCs adhered to plastic, expressed classical markers and differentiated into bone and fat lineages in a similar manner to BM-MSCs. Furthermore, using a micro (mi)RNA array we showed that they were 64% homologous with a similar core subset of miRNAs (Lindsay et al., 2016). We and others have also shown that while they were identical in their expression of a panel of CD markers, a greater proportion of OM-MSCs expressed nestin immunoreactivity; 100% of OM-MSCs express nestin compared to around 50% of BM-MSCs (Lindsay et al., 2010, Johnstone et al., 2015, Delorme et al., 2010, Fig. 2, Table 1). The relevance of nestin-positive MSCs within the BM is now being evaluated by researchers. Nestin is a class VI intermediate filament protein which was originally identified as a stem cell marker for neuroepithelial cells (Lendah et al., 1990), although, it has been reported to label a range of cells from neural stem cells, fibroblasts and reactive glia (Kishaba et al., 2010, Toft et al., 2013, Xie et al., 2015). Table 1 summarises the comparative differences reported to date on OM-MSCs and BM-MSCs. Since OM-MSCs have only recently been identified, very few direct comparisons of their biological properties to BM-MSCs have been reported. Although, we have directly compared the two types of MSCs abilty to promote CNS myelination *in vitro* in the table, comparative data on myelination potential *in vivo* is limited. BM-MSCs have been shown to increase the number of oligodendrocytes, and enhance remyelination in EAE but similar data for OM-MSCs has not been published (see review of Cohen, 2013).

**Fig. 2:**
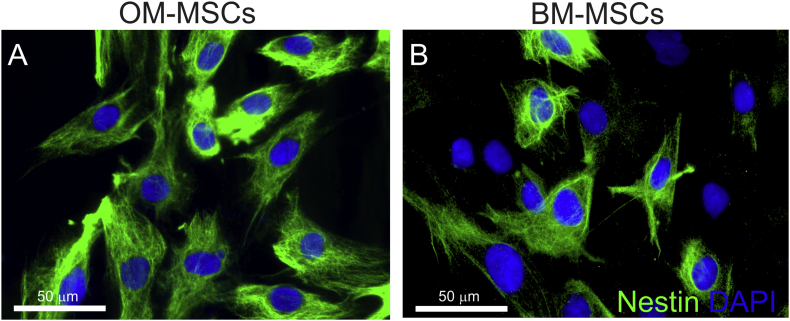
Differential expression of nestin on OM-MSCs and BM-MSCs (nestin stained green; nuclei stained blue DAPI). Scale bar represents 50 μm. (For interpretation of the references to colour in this figure legend, the reader is referred to the web version of this article.)

**Table 1:** Comparison of biological properties of olfactory mucosal- and bone marrow-derived MSCs.

Characteristics	OM-MSCs	BM-MSCs	Reference
**Marker expression**			
Express most classical MSC markers with difference reported only in:			
Nestin	+++	+	Delorme et al., 2010, Lindsay et al., 2013, Di Trapani et al., 2013, Johnstone et al., 2015, Rui et al., 2016
CD271	+	+	Lindsay et al., 2013, Johnstone et al., 2015
CD200, CD146	−	+	Delorme et al., 2010, Wetzig et al., 2013
**Micro RNA expression**			
miR-differentially expressed:			Lindsay et al., 2016, Tomé et al., 2011
140-5p; 10b-5p; 335-5p; 3665; 3188; 2861; 4281; 762; 874; 1915; 638; 424-5p; 140-5p; 224-5p; 140-3p; 939; 1225-5p.	−	+	
4291; 20a-5p; 25-3p; 106b-5p; 301a-3p; 195-5p; 497-5p; 93-5p; 3529-3p; 146a-5p	+	−	
**Gene expression**			
Nestin, CXCL12, CD90, S100A4, CD40	+++	+	Delorme et al., 2010, Johnstone et al., 2015; Lindsay et al., 2016;Wetzig et al., 2013
CD73	+	+++	Johnstone et al., 2015
**Chemokines**			
CXCL12	+++	+	Lindsay et al., 2016
IL-6, IL-8, CCL2, IL-10, TGF-β	+	+++	Lindsay et al., 2016;Rui et al., 2016
**Biological properties**			
Clonal efficiency, Proliferation	+++	−	Delorme et al., 2010, Lindsay et al., 2013
Osteogenesis, Adipogenesis	+	+++	Delorme et al., 2010, Lindsay et al., 2013, Wetzig et al., 2013
Chondrogenesis	−	+++	Delorme et al., 2010, Lindsay et al., 2013, Wetzig et al., 2013
T-cell proliferation	−−−	−	Di Trapani et al., 2013, Rui et al., 2016
CNS Myelination (*in vitro*)	+++	+/−	Lindsay et al., 2013; Lindsay et al., 2016

### 1.3 Nestin-positive OM- and BM-MSCs

**Fig. 3:**
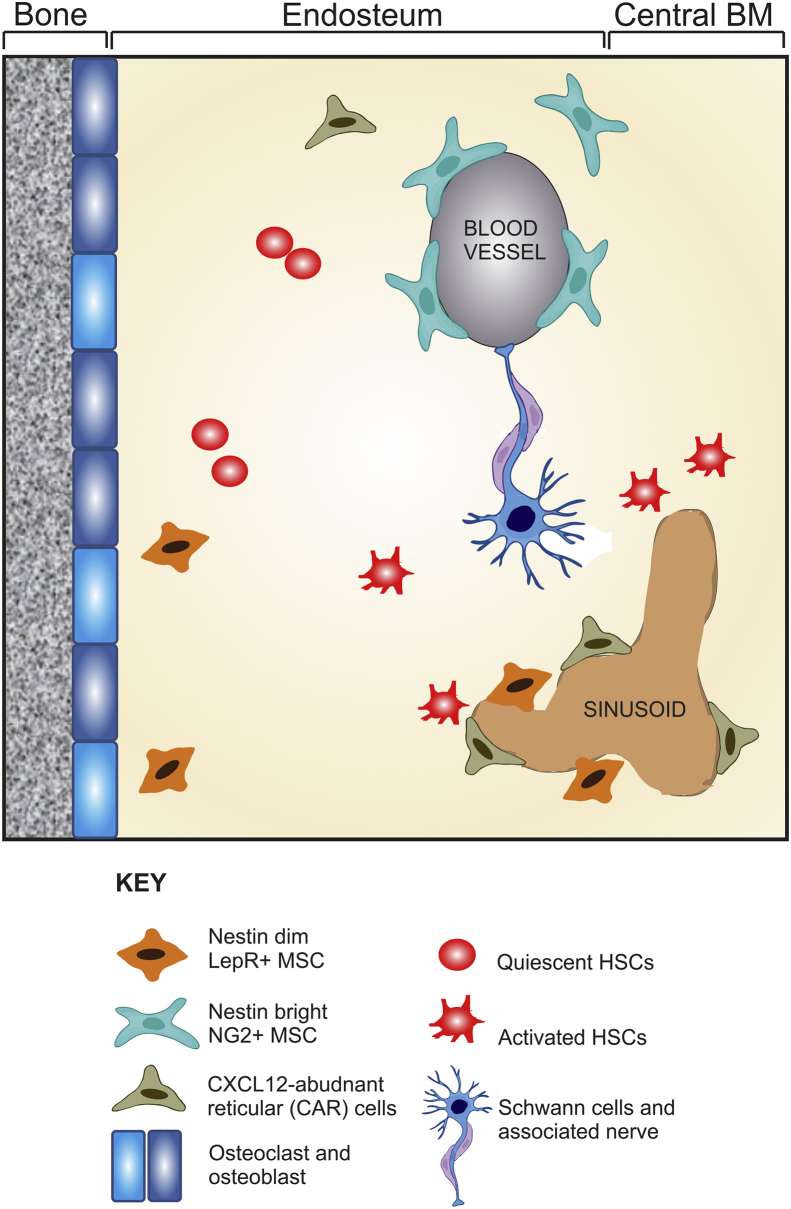
Schematic illustrating the various cell types in the rodent bone marrow niche including the range of cells secreting CXCL12 based on Boulais and Frenette, 2015.

Interestingly, a subpopulation of BM-MSCs have also been reported to express nestin (Tondreau et al., 2004, Wiese et al., 2004) and more detailed studies demonstrated that the nestin-positive MSCs are similar to early progenitor cells that are able to self-renew and differentiate into bone, fat and adipose (Mendez-Ferrer et al., 2010). These early progenitors have been hypothesised to be “mesodermal progenitor cells” or MPCs by other researchers (Petrini et al., 2009; Pacini and Petrini, 2014). The nestin-positive MSCs have been shown to co-localize with HSCs supporting their maintenance and homing (Isern and Mendez-Ferrer, 2011). Using transgenic mice that express the regulatory elements of the nestin-promotor (Nes-GFP) it was demonstrated that the nestin-positive MSC subpopulation originate from the neural crest and have special HSC niche functions, while the nestin-negative MSCs originate from the mesoderm and give rise to bone and cartilage (Isern et al., 2014). Other epithelial tissues have also been suggested to contain neural crest derived mesenchymal progenitors including the human oral mucosa (Davies et al., 2010), oral gingivae (Xu et al., 2013), dental pulp tissue (Volponi et al., 2010) and airway epithelium (Ortega-Martínez et al., 2015). Furthermore, nestin-positive MSCs were identified in airway epithelium within the perivascular areas and in connective tissue that is in close proximity to the airway epithelium (Ortega-Martínez et al., 2015). These authors suggest that these MSCs circulate in the bloodstream, transmigrate through blood vessels and localize in the epithelium to participate in its turnover by being able to generate several different types of lung tissues. This could be a general feature of many different types of mucosa that exist throughout the body, where rapid turnover of cells is required after damage or during normal cell turnover. The importance of isolating nestin-positive neural crest derived MSCs for therapy over nestin-negative MSCs is not yet fully known. Moreover, the various subtypes of MSCs that make up the BM niche (illustrated in Fig. 3) have been mainly identified from rodent studies, and it is yet not known if similar cells exist in the human BM; although several recent reports have shown that the human BM niche contains nestin-positive MSCs (Crisan et al., 2008, Matsuoka et al., 2015, Pacini and Petrini, 2014, Schajnovitz et al., 2011). These data corroborate our own published results which demonstrate that around 50% of purified BM-MSC express nestin immunoreactivity (Johnstone et al., 2015).

We have shown that OM-MSCs, which are 100% nestin-positive better promoted *in vitro* CNS myelination when compared to BM-MSCs (Lindsay et al., 2013) and that they also moved microglia to an anti-inflammatory phenotype, which correlated with their secretion of less pro-inflammatory cytokines (Lindsay et al., 2016). In addition, others have shown different differentiation capabilities of nestin-positive neural crest derived MSCs, and have shown that they too have beneficial immunoregulatory function over immune cells when compared to mesodermal derived MSCs (Xu et al., 2013). Therefore, it may be that there exists a subpopulation of neural crest derived MSCs in a variety of different tissues that are more suited to regenerative medicine.

### 1.4 The role of CXCL12 in the bone marrow niche

The BM contains many cell types including HSCs, endothelial cells, osteoblasts, osteoprogenitors, chemokine (C–X–C motif) ligand 12 (CXCL12)-abundant reticular (CAR) cells and nestin+ NG2+ MSCs (which have been classified primarily based on the availability of a specific Cre-recombinase mouse or related reporter mouse which include *Lepr*-Cre (Ding et al., 2012), *Prx1*-Cre (Greenbaum et al., 2013), *Nestin*-Cre (Isern et al., 2014, Mendez-Ferrer et al., 2010), CXCL12-GFP (Omatsu et al., 2010; Sugiyama and Nagasawa, 2012). Cells were thus identified as nestin-GFP^dim^ and nestin-GFP^bright^ in one tracing experiment (Isern and Mendez-Ferrer, 2011) and nestin-LepR+ MSCs from a different reporter mouse. Moreover, definition of these various cell types does not exclude overlapping phenotype between each of these classified populations and so none of the defined cell types are exclusive from each other (Hoggatt et al., 2016, Coste et al., 2015, Ding et al., 2012, Greenbaum et al., 2013, Sugiyama and Nagasawa, 2012). It is well understood that CXCL12 (stromal cell-derived factor-1) plays an important role within the human and rodent BM in controlling HSC and progenitor function (Isern et al., 2014, Schajnovitz et al., 2011). Our own investigations have also revealed an important role for CXCL12 in human OM-MSC biology. OM-MSCs under the regulation of miR140-5p secrete more CXCL12 than BM-MSCs which is responsible for promoting rat CNS myelination *in vitro* to a greater extent than BM-MSCs (Lindsay et al., 2013, Lindsay et al., 2016). In the BM, CXCL12 has a range of HSC regulatory functions from retention (blocking their differentiation and migration) to quiescence and repopulation activity (Greenbaum et al., 2013, Schajnovitz et al., 2011). BM-MSCs take part in the host defence by rapidly releasing CXCL12 into the circulation, which results in the mobilisation of progenitor cells (Kollet et al., 2003, Petit et al., 2002).

In the mouse BM three perivascular stromal cell populations that express high levels of CXCL12 have been identified: CXCL12-abundant reticular (CAR) cells, nestin-GFP+ stromal cells, and lepR+ stromal cells (Fig. 3). These stromal cell populations are defined by transgene expression using defined stromal-specific promoters, and as discussed above, it is likely that there is considerable overlap (Anthony and Link, 2014). Other cells within the BM have been shown to secrete CXCL12 including, osteoblasts, and endothelial cells (Ding et al., 2012, Sugiyama and Nagasawa, 2012). However, more recent detailed analysis using cell type specific promoters to knock out CXCL12 expression has shown that HSC maintenance and mobilisation was dependent upon nestin-positive mesenchymal progenitors (Greenbaum et al., 2013). Indeed deletion of nestin positive MSCs from the bone marrow has been shown to cause a dramatic expansion of HSCs due to a reduction in CXCL12 levels (Arranz et al., 2014). What this data highlights is an important role for CXCL12 secretion by nestin-positive MSCs and that such MSCs are central participants in HSC regulation.

It is also thought that within the BM microenvironment populations of cells exist which have differing roles in regulating the biology of HSCs (Boulais and Frenette, 2015, Coste et al., 2015). The endosteal niche is mainly composed of osteoblasts, osteoclasts, adipocytes, CAR cells, stromal cells, nestin+ MSCs, which together maintain HSCs in a quiescent state. In contrast, the vascular niche which is located near to BM sinusoid vessels is composed of perivascular (nestin+) stromal cells, CAR cells and the peripheral glia, Schwann cells. The former two cell types favouring HSC activation and recruitment, with the latter involved in HSC maintenance. Interestingly, there has been a report to suggest that human OM-MSCs can also promote the *in vitro* survival, proliferation and differentiation of human HSCs (Diaz-Solano et al., 2012). Thus it is possible then that nestin+ MSCs from the OM are similar to these nestin+ MSCs from the BM or are in fact the same neural crest derived source of MSCs. Further experiments, including fate mapping are required to fully elucidate whether OM-MSCs are indeed neural crest derived although it is tempting to speculate that they are, as it has been shown using reporter mice, that olfactory ensheathing cells (Barraud et al., 2010), olfactory receptor neurons, sustentacular cells and the basal stem cells from the olfactory epithelium after injury, originate from the neural crest (Suzuki et al., 2013).

### 1.5 MSCs for the treatment of CNS disease/injury

BM-MSCs have long been considered as candidates for the transplant-mediated repair of the injured or diseased CNS. They have already been evaluated in a number of clinical trials for the treatment of a range of CNS disorders including amyotrophic lateral sclerosis (Mazzini et al., 2003, Mazzini et al., 2009, Mazzini et al., 2012 Oh and Nör, 2015); progressive supranuclear palsy (Giordano et al., 2014); cerebral palsy in children (Wang et al., 2013); ischaemic heart failure (Mathiasen et al., 2015, Rodrigo et al., 2013); myocardial infarction (Hare et al., 2009); knee osteoarthritis (Vega et al., 2015); acute respiratory distress syndrome (Wilson et al., 2015) and Crohn's disease (Duijvestein et al., 2010). However most of these studies are phase I and have only shown safety of administration rather than efficacy.

Since MSCs are thought to have an immunomodulatory role, they have also been proposed for the treatment of the immune mediated demyelinating disease, Multiple Sclerosis (MS). In experimental autoimmune encephalomyelitis (EAE), an animal model of MS, MSC transplantation has been shown to have promising beneficial effects, using both syngeneic cells (Gerdoni et al., 2007, Karussis et al., 2008) as well as human cells (Bai et al., 2009, Rafei et al., 2009). These include a reduction in demyelination, axonal loss, leucocyte infiltration, inhibition of T cell proliferation, and macrophage/microglia activation. The beneficial effects of BM-MSC transplantation in EAE are generally attributed to immunomodulatory effects in the periphery, although they may also migrate into the CNS to secrete soluble neuroprotective factors that support endogenous tissue repair and remyelination (Bai et al., 2009, Freedman et al., 2010, Pluchino and Martino, 2008). Culturing BM-MSCs with microglia has indicated that they promote a switch in LPS-activated microglia from a detrimental phenotype to one more beneficial (Giunti et al., 2012). Moreover, promising effects have been seen in human trials of autologous transplantation of BM-MSCs in secondary progressive patients illustrating their safety. There is also evidence of neuroprotection from structural, functional, and physiological improvement which strongly supports their future use as treatment in MS (Bonab et al., 2012, Connick et al., 2012, Harris et al., 2012, Uccelli et al., 2013; Llufriu et al., 2014).

While it has been known for numerous years that the human olfactory tissue contains multipotent stem cells which were typically from the basal layer in the epithelium, or from the olfactory bulb (Goldstein et al., 1968; Roisen et al., 2001; Murrell et al., 2005, 2008; Marshall et al., 2006), it was only recently that mesenchymal like stromal cells were identified from the lamina propria of the OM (Tomé et al., 2009, Delorme et al., 2010), and since then OM derived MSCs have only been described in a handful of reports detailing their use in tissue repair. For example, a study was carried out using human MSCs from the OM in a mouse model of early onset sensorineural hearing loss (Pandit et al., 2011). Here olfactory MSCs, prepared as described by Delorme et al. (2010), were injected into the cochlea of A/J mice which exhibit early-onset progressive sensorineural hearing loss. OM-MSC transplantation was seen to prevent hearing loss as assessed by response to both click and pure tone stimuli suggesting they protect against loss of auditory function. Other studies have been reported on the repair potential of OM-MSCs using mice with hippocampal lesions created by the injection of a NMDA agonist. In these mice, transplantation of OM-MSCs restored learning and memory using behavioural testing when compared to non-transplanted control mice (Nivet et al., 2011). A recent study using mouse derived OM-MSCs also demonstrated that they could suppress arthritis onset in a mouse model of collagen-induced arthritis (Rui et al., 2016).

Furthermore, it is possible that in experiments which use dissociated olfactory mucosa cells, OM-MSCs may be present in the heterogeneous cell mix (Lima et al., 2006, Lima et al., 2010 Murrell et al., 2008, Chhabra et al., 2009, Granger et al., 2012, Iwatsuki et al., 2016, Wang et al., 2016). However, there is no detailed analysis of the cellular composition of these cellular mixes or which cell type(s) dominate or in fact which cell type has reparative properties. Indeed a spinal cord injured patient who received a transplant of un-purified olfactory mucosa was found to develop an intramedullary spinal cord mass 8 years post-surgery, composed of cysts lined with respiratory epithelium (Dlouhy et al., 2014). Therefore, the use of un-purified olfactory mucosal cells cannot be directly compared to purified OM-MSCs and indeed highlights the importance of transplanting cells which are fully characterised. In terms of therapeutic paradigms it is vital that any cell type that is to be transplanted is fully characterised. Unfortunately, due to the technical variability in harvesting and growing cells from both BM and OM it is difficult to compare data across experiments. However, from the limited studies and our own data, which demonstrates OM-MSCs promote rat CNS myelination *in vitro* (Lindsay et al., 2010, Lindsay et al., 2016) and *in vivo* (Lindsay et al., submitted), we would postulate they may be an alternative candidate for the treatment in MS or other demyelinating diseases/injuries. Similarly they may be advantageous where neuroprotection or neurite outgrowth is important for repair eg retinitis pigmentosa, age-related macular degeneration, ALS, and stroke. The identification that OM-MSCs secrete higher levels of the chemokine CXCL12 than BM-MSCs (Lindsay et al., 2016), which we and others have shown to be important in augmenting endogenous myelination (Maysami et al., 2006, Lindsay et al., 2013, Lindsay et al., 2016 Zilkha-Falb et al., 2016) also adds to the desirability as a candidate cell for transplantation. Lastly as MSCs are known to home to damaged tissue (Laroni et al., 2013, Eseonu and De Bari, 2015) and secrete a plethora of pro-repair factors, their use as a treatment is more beneficial than application of a chemokine alone (Allers et al., 2014, Joyce et al., 2010, Laroni et al., 2013).

In conclusion, we feel that nestin-positive OM-MSCs may be derived from the neural crest, and are an easily accessible source of cells which may be therapeutically advantageous over BM-MSCs for the treatment of demyelinating conditions such as MS.
